# Advances in Ginsenosides

**DOI:** 10.3390/biom10050681

**Published:** 2020-04-28

**Authors:** Jen-Tsung Chen

**Affiliations:** Department of Life Sciences, National University of Kaohsiung, Kaohsiung 811, Taiwan; jentsung@nuk.edu.tw

Ginsenosides are unique to plants that belong to the *Panax* genus. The genus name *Panax* means “all-curing” in Greek, and one of the representative plants, Asian ginseng (*Panax ginseng* Meyer), has been classified as a top-grade herb in traditional Chinese medicine according to the Divine Husbandman’s Herbal Foundation Canon. It has been used as medicine as well as a tonic for over two thousand years and is still popular with increasing demand in oriental countries nowadays. It has long been recognized that ginseng remedies possess a wide range of benefits for human health. However, their bioactivities are diverse, interconnective, and complicated that need to be clarified. Hence, this special issue attempts to explore new insights into characterization, quantitation, production, biotechnology as well as bioactivities of ginsenosides.

## 1. Characterization and Quantitation

Up to date, there are more than one hundred ginsenosides have been identified. Jeon et al. reported a new protopanaxatriol-type ginsenoside, namely ginsenoside MT1, that occasionally obtained from enzymatic converted products derived from ginsenoside Re [1]. They suggested potential commercial applications of MT1 with nutraceutical, pharmaceutical or cosmeceutical values, and further tests for the pharmacological effects are ongoing. Chen et al. profiled ginsenoside components from various parts of Asian ginseng (*P. ginseng* Meyer) and American ginseng (*P. quinquefolium* L.) that grown in the North Island of New Zealand mainly using liquid chromatography-tandem mass spectrometry (LC-MS/MS) analysis [2]. They found these two species of ginseng have a similar number and total amounts of ginsenosides. Yoon et al. profiled primary metabolites and ginsenoside constituents of *P. ginseng* berries from seven cultivars using high-resolution magic-angle-spinning nuclear magnetic resonance (HR-MAS NMR) spectroscopy and ultra-performance liquid chromatography-quadrupole-time-of-flight mass spectrometry (UPLC-QTOF/MS), respectively [3]. They confirmed that a characteristic metabolic pattern presented in the cultivar Gopoong, and a differential content of 20(S)-protopanaxatriol (PPT)-type ginsenosides was found in the cultivars Kumpoong and Sunwon.

## 2. Production and Biotechnology

In the ginseng extract, ginsenoside Rg3(S) only presents a limited amount and its production could be enhanced conventionally by the steaming method and heat acid treatment but some inevitable by-products were also produced in the meantime. Siddiqi et al. established an alternative way using a novel glycoside hydrolase for the enzymatic conversion of ginsenoside Rg3(S) from ginsenosides Rb1 and Rd [4]. The enzyme originated from *Lactobacillus ginsenosidimutans* EMML 3041^T^ that exited in Korean fermented pickle (kimchi), and subsequent has been modified into a recombinant enzyme, namely BglL.gin-952. According to the results, the authors proposed that BglL.gin-952 has a high ability of biotransformation that could be applied in the industrial level production of ginsenoside Rg3(S) in the future. Gantait et al. summarized the approaches of in vitro plant biotechnology for ginsenosides production [5]. It is an intensive topic that many tools that have been reported for this purpose, including bioreactor, genetic transformation, in vitro mutagenesis, hairy root culture, polyploidy induction, as well as conventional tissue-culture protocols such as adventitious root culture, callus culture, cell suspension, direct organogenesis, liquid culture, protoplast culture, and somatic embryogenesis, together with the utilization of additives, elicitors, and precursors.

## 3. Bioactivities

Epithelial–mesenchymal transition (EMT) is crucial for differentiation and development, and it is also a key step in cancer metastasis. Wang et al. studied the effect of the (20S)ginsenoside Rh2 (G-Rh2) on EMT, and they concluded that in a human breast cancer cell line, G-Rh2 could inhibit the NF-κB activation as well as the related EMT by targeting an NF-κB p50 subunit binding protein annexin A2 [6].

It has been recognized that adult neurogenesis in the hippocampus is the main contributor to prevent age-related cognitive impairments as well as neurodegenerative diseases. Oh et al. investigated the effect of compound K (CK), a product from ginsenosides Rb1, Rb2, and Rc through intestinal microbial conversion, on hippocampal neurogenesis in mice [7]. They found CK promoted cell proliferation in the dentate gyrus of both young and elderly mice. Hence, the authors proposed that CK has the potential benefit against neurodegenerative disorders such as Alzheimer’s disease.

Ischemic stroke is a serious cerebrovascular disease and it is one of the leading causes of death worldwide. Xie et al. studied the bioactivity of notoginseng leaf triterpenes (PNGL) on cerebral ischemia and reperfusion injury of ischemic stroke [8]. The results showed that PNGL decreased the expression of the high mobility group box-1 protein (HMGB1) and consequently inhibited HMGB1-triggered inflammation. In addition, a reduction of several pro-inflammatory mediators, as well as a suppression of the activation of MAPKs and NF-κB signaling pathways, were found in the presence of PNGL.

In autoimmune diseases, IL-17A-producing CD4^+^ T cells (Th17) were recognized as critically pathogenic immune cells that mediate tissue inflammation. Park et al. demonstrated that ginsenoside Rg3 inhibited Th17 differentiation as well as Th17-mediated neuro-inflammation in a dendritic cell-T co-culture system [9]. The authors suggested that Rg3 could be a potential agent for treating certain autoimmune disorders driven by Th17 cells.

Allergies including allergic rhinitis, anaphylaxis, asthma, atopic conjunctivitis, atopic dermatitis, pruritus, and so on, are common disorders worldwide with prevalence ranges from 10% to 40% population. Han and Kim summarized the effects of white ginseng (WG, dried root of *Panax* sp.), red ginseng (RG, steamed and dried root of *Panax* sp.), and fermented WG and RG (fWG and fRG) on allergic disorders [10]. Overall, the extract of fRG and ginsenosides CK, Rh1, and Rh2 have better potential than other ginseng remedies to alleviate allergic disorders.

Inflammation is part of immunity associated with diseases and physiological conditions. Im refined the knowledge of ginsenosides for anti-inflammation and summarized the action mechanism [11]. It was proposed that ginsenosides exert their anti-inflammation actions through retarding expressions of pro-inflammatory cytokines, TNF-α, IL-1β, and IL-6, and enzymes, iNOS, and COX-2, in M1-polarized macrophages. Besides, it was mentioned that a new focusing on the pro-resolving effect of ginsenosides in M2-polarized macrophages is emerging.

Theoretically, the understanding of molecular mechanisms involved in ligand–protein interactions is crucial for drug design. Guo et al. investigated the chirality of C20-24 epimeric epoxides that transformed from protopanaxadiol-type ginsenosides [12]. Through the analysis of molecular docking, the authors confirmed consistency in some previous cell/animal-based experimental findings and gain insights into the stereo-selectivity of ginsenosides. Their findings could support the rational development of ginseng products in the future.

An overview of the *P. ginseng* pharmacopuncture was reported by Lee et al. to systematically refine safety, physiological responses, and clinical effects regarding the manufacturing processes [13]. The authors concluded that the process should be standardized including a comprehensive comparison of the quantity and quality of ginsenoside components of injections for improving clinical outcomes in the future.

## 4. Conclusions and Perspectives

This special issue collected recent innovative research and review articles on analytical techniques, production protocols, biotechnological tools, and new insights into bioactivities of ginsenosides including the effects on epithelial–mesenchymal transition, hippocampal neurogenesis and inflammation as well as on diseases such as ischemic stroke, autoimmune diseases, and allergic disorders. In addition, the analysis through molecular docking and an overview of the *P. ginseng* pharmacopuncture were also included. With the rapid development of molecular tools such as high-throughput technologies and integrative multi-omics, there are more opportunities for scientists to clarify the efficacy of ginseng products and the ginsenoside components in the future.
